# Mirvetuximab soravtansine in platinum-resistant ovarian cancer: from biological rationale to clinical impact and future directions

**DOI:** 10.1007/s10147-026-03090-3

**Published:** 2026-06-13

**Authors:** Seiji Mabuchi, Ai Kusakabe, Tomoyuki Sasano

**Affiliations:** Department of Obstetrics and Gynecology, Hyogo Medical University, 1-1, Mukogawa-Cho, Nishinomiya, Hyogo 663-8501 Japan

**Keywords:** Mirvetuximab soravtansine, Folate receptor alpha, Platinum-resistant ovarian cancer, Antibody–drug conjugate, Biomarker-driven therapy

## Abstract

Platinum-resistant ovarian cancer (PROC) remains a major clinical challenge due to limited therapeutic options and poor prognosis. Conventional treatments provide only modest benefits, and immune checkpoint inhibitors have shown limited efficacy in unselected populations, underscoring the need for biomarker-driven strategies. Mirvetuximab soravtansine (MIRV) is a first-in-class antibody–drug conjugate targeting folate receptor alpha (FRα), which is highly expressed in a substantial proportion of epithelial ovarian cancers. By combining selective tumor targeting with intracellular delivery of the cytotoxic payload DM4, MIRV achieves potent antitumor activity with a manageable safety profile and evidence of a clinically relevant bystander effect. Clinical trials including SORAYA and MIRASOL demonstrated that MIRV significantly improved response rates, progression-free survival, and overall survival in patients with FRα-high PROC, thereby establishing MIRV as a clinically meaningful treatment option. Ongoing research is evaluating MIRV by using combination regimens, earlier treatment lines, and maintenance strategies. Despite these advances, challenges remain, including resistance mechanisms, optimization of biomarker assessments, and cost considerations. Future efforts should focus on refined patient selection, rational combination strategies, and next-generation antibody–drug conjugates, which are expected to further expand the clinical utility of FRα-targeted therapy.

## Introduction

Epithelial ovarian cancer (EOC) remains the most lethal gynecological malignancy worldwide, largely due to late-stage diagnosis and frequent recurrence. Although most patients initially respond to cytoreductive surgery and platinum-based chemotherapy, up to 80% eventually relapse and many patients develop platinum-resistant ovarian cancer (PROC), defined as progression within six months of platinum-based therapy [1, 2]. The prognosis of PROC remains poor, with the standard of care treatment relying on sequential non-platinum chemotherapy, with or without bevacizumab, yielding low response rates (< 15%) and limited progression-free survival (PFS) [1, 2]. Secondary debulking surgery and radiotherapy offer minimal benefit, and immune checkpoint inhibitors have shown limited efficacy in unselected populations [2–4], highlighting the need for novel biomarker-based therapies.

Antibody–drug conjugates (ADCs) have emerged as a promising therapeutic class combining targeted delivery with potent cytotoxicity [5]. Mirvetuximab soravtansine (MIRV) is a first-in-class ADC targeting folate receptor alpha (FRα), which is highly expressed in EOC with limited normal tissue distribution [6–9]. MIRV consists of a humanized anti-FRα antibody linked to the maytansinoid payload DM4, which disrupts microtubule dynamics following internalization [10].

The development of MIRV represents a shift toward biomarker-selected therapies in ovarian cancer. Early-phase studies demonstrated encouraging activity in FRα-positive PROC [11]. Although the phase III FORWARD I trial did not meet its primary endpoint, subsequent analyses emphasized the importance of high FRα expression and optimized diagnostic assays [12]. These insights led to the SORAYA and MIRASOL trials, which confirmed improved response rates, PFS, and overall survival (OS) compared with standard chemotherapy [13–15]. MIRV is approved in the United States and Europe, with approval in Japan anticipated in the near future.

This review summarizes the biological rationale, mechanism of action, and clinical evidence for MIRV as well as its safety profile, limitations, and future directions in the treatment of ovarian cancer.

## Biological rationale for targeting FRα in ovarian cancer

### FRα biology and function

Folate, a water-soluble B vitamin, is essential for DNA synthesis and cellular proliferation through one-carbon metabolism, nucleotide biosynthesis, and DNA methylation [1]. The folate receptor family consists of four isoforms—FRα, FRβ, FRγ, and FRδ—with distinct distributions and functions. Among these, FRα, encoded by the FOLR1 gene, is the most studied in cancer. FRα is a glycosylphosphatidylinositol (GPI)-anchored cell surface protein that binds folate with high affinity and mediates uptake via receptor-mediated endocytosis [16, 17]. After internalization, folate contributes to nucleotide biosynthesis and methylation processes required for tumor growth [18, 19].

Under physiological conditions, FRα expression is tightly restricted and largely confined to the apical surface of polarized epithelial cells, limiting accessibility to circulating ligands and therapeutic agents. In contrast, malignant transformation disrupts epithelial polarity, redistributing FRα to surfaces exposed to the bloodstream [16, 17]. This increases accessibility to systemically administered therapies. Thus, tumor-selective expression, surface accessibility, and rapid internalization establish FRα as a rational target for drug delivery rather than direct inhibition.

### FRα expression and its prognostic significance in ovarian cancer

FRα is expressed in various epithelial malignancies, including ovarian, endometrial, lung, breast, and gastrointestinal cancers, with variable rates [6–9, 16–20]. In ovarian cancer, particularly high-grade serous carcinoma, FRα expression is frequent, relatively homogeneous, and mainly localized to the cell surface, facilitating targeting and internalization [6–9, 16–20].

FRα expression appears relatively stable over the disease course and is generally maintained after chemotherapy. Analyses of paired tumor samples obtained at diagnosis and recurrence demonstrated minimal changes in FRα expression, supporting the use of archival tissue for biomarker assessment in recurrent disease [8, 16, 20–22]. However, spatial and temporal heterogeneity may still occur in some tumors, including variability between primary and metastatic lesions, which may potentially influence treatment response [23].

The prognostic significance of FRα remains uncertain. Some studies suggest associations with aggressive clinicopathological features, whereas others show no consistent relationship with survival outcomes [6, 17, 21]. Overall, the high prevalence, relative homogeneity, and relative stability of FRα expression support its role as a predictive biomarker and therapeutic target.

### FRα as a therapeutic target

FRα has features that make it an attractive therapeutic target, particularly for drug delivery approaches, including restricted expression in normal tissues, high tumor accessibility, and efficient ligand-induced internalization [10]. Although not an oncogenic driver, FRα functions as a folate transporter supporting tumor proliferation. Thus, direct receptor inhibition is unlikely to produce substantial antitumor effects. Instead, its clinical value lies in serving as an accessible, internalizing surface antigen for targeted cytotoxic delivery.

**Fig. 1:**
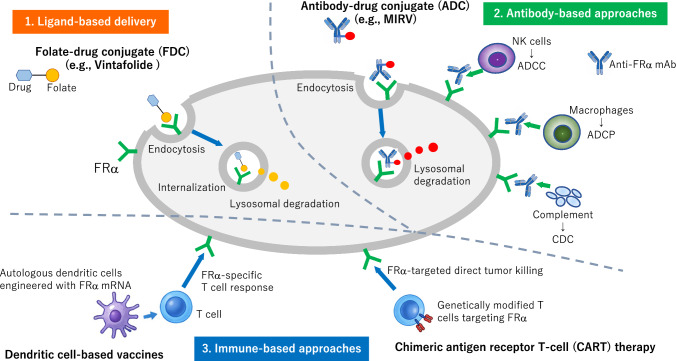
FRα-targeted therapeutic strategies. FRα-targeted therapies can be broadly categorized into three mechanistically distinct but complementary approaches. (1) Ligand-based delivery systems utilize folate or folate analogs to selectively deliver cytotoxic agents into FRα-expressing tumor cells via receptor-mediated internalization. This category includes folate–drug conjugates (FDCs) and small molecule–drug conjugates (SMDCs), which exploit the high affinity of folate for FRα to achieve targeted intracellular drug release. (2) Antibody-based approaches include monoclonal antibodies and antibody–drug conjugates (ADCs). Monoclonal antibodies can exert direct antitumor effects or induce immune-mediated cytotoxicity through mechanisms such as antibody-dependent cellular cytotoxicity (ADCC), antibody-dependent cellular phagocytosis (ADCP), and complement-dependent cytotoxicity (CDC). ADCs further enable the intracellular delivery of potent cytotoxic payloads following antibody binding and receptor-mediated internalization. (3) Immune-based approaches aim to activate or enhance antitumor immune responses against FRα-expressing cells. These include dendritic cell–based vaccines engineered with FRα mRNA to induce FRα-specific T cell responses, as well as chimeric antigen receptor (CAR) T cells designed to directly recognize and eliminate FRα-expressing tumor cells. Collectively, these three categories provide a unified framework that integrates previously reported FRα-targeted therapeutic modalities, highlighting FRα as a tumor-selective surface antigen that can be exploited for targeted drug delivery and immune-based therapies.

FRα-targeted strategies can be grouped into three major approaches, as summarized in Fig. 1. First, ligand-based systems, such as folate–drug conjugates (FDCs), use folate or analogs to deliver cytotoxic payloads into FRα-expressing cells. Second, antibody-based approaches, including monoclonal antibodies and antibody–drug conjugates (ADCs), exploit antibody specificity for targeted delivery. Monoclonal antibodies may also induce cytotoxicity via ADCC, ADCP, and CDC, while ADCs enable intracellular delivery of potent agents. Third, immune-based strategies, such as vaccines and CAR T cells, enhance antitumor immune responses against FRα-expressing cells.

### Lessons from earlier FRα-targeted therapies

Earlier FRα-targeted therapies, including vintafolide and farletuzumab, failed to demonstrate clinical benefit in phase III trials [2, 24–26], highlighting limitations of first-generation strategies. Vintafolide, a ligand-based folate–drug conjugate, relied on FRα-mediated uptake but showed limited efficacy, likely due to insufficient payload potency and suboptimal patient selection. Farletuzumab, an antibody-based therapy designed to induce immune-mediated cytotoxicity via ADCC and CDC [25, 26], showed modest activity, largely limited to disease stabilization.

These findings highlight two challenges: FRα targeting without a sufficiently potent cytotoxic payload is unlikely to achieve meaningful tumor regression, and reliance on immune-mediated mechanisms may be affected by variability in host immunity and the tumor microenvironment. In this context, ADCs represent a rational evolution, enabling direct tumor cell killing through intracellular delivery of highly potent cytotoxic agents, thereby contributing to the success of next-generation agents such as MIRV [13–15].

## Mechanism of action of mirvetuximab soravtansine (MIRV)

### Structure of mirvetuximab soravtansine

**Fig. 2:**
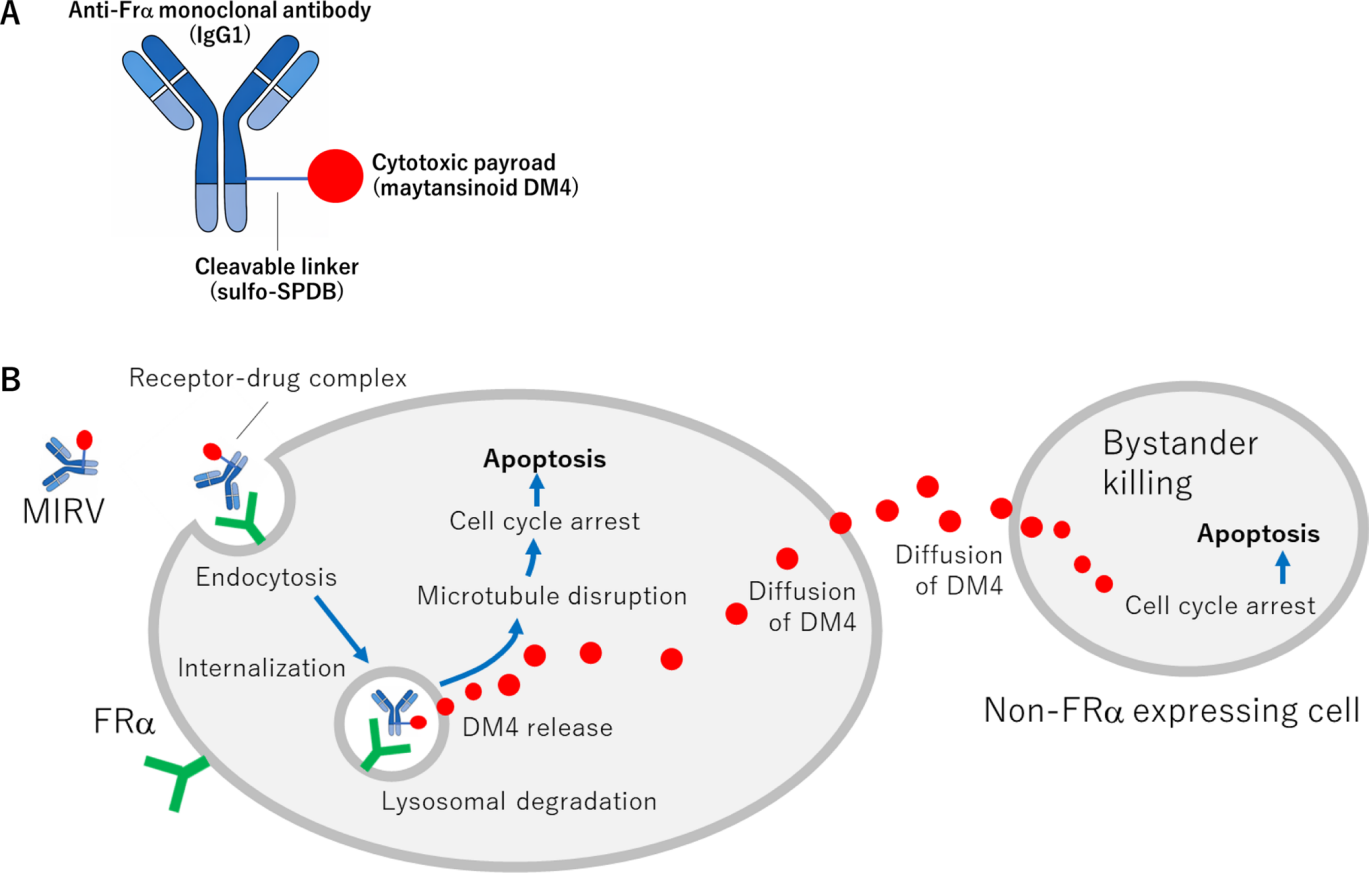
Structure and mechanism of action of mirvetuximab soravtansine (MIRV). **A** MIRV consists of a humanized anti–folate receptor alpha (FRα) monoclonal antibody linked via a cleavable disulfide linker to the maytansinoid payload DM4 (N2′-deacetyl-N2′-(4-mercapto-4-methyl-1-oxopentyl)-maytansine), a potent microtubule inhibitor. **B** Upon binding to FRα on the tumor cell surface, MIRV is internalized via receptor-mediated endocytosis and trafficked to lysosomes, where linker cleavage releases DM4 and its active metabolites. These metabolites bind to tubulin and disrupt microtubule dynamics, leading to cell cycle arrest and apoptosis. Membrane-permeable DM4 metabolites can also diffuse into neighboring tumor cells, including those with low or absent FRα expression, resulting in bystander cytotoxic effects that may enhance antitumor activity in tumors with heterogeneous FRα expression

MIRV consists of three key components (Fig. 2A): a humanized anti-FRα monoclonal antibody, a cleavable disulfide linker, and the maytansinoid payload DM4 (N2′-deacetyl-N2′-(4-mercapto-4-methyl-1-oxopentyl)-maytansine), a potent microtubule inhibitor [10]. The antibody enables selective binding to FRα-expressing tumor cells, while the linker is designed to remain stable in circulation and undergo intracellular cleavage to release the cytotoxic payload.

### Cellular uptake, intracellular processing, and cytotoxic effects

Following systemic administration, MIRV binds with high affinity to FRα on the tumor cell surface and is internalized via receptor-mediated endocytosis [10]. The intracellular trafficking and cytotoxic mechanism of MIRV are summarized in Fig. 2B. Efficient internalization is a critical determinant of ADC activity. Once internalized, MIRV is trafficked to lysosomes, where cleavage of the disulfide linker releases DM4 and its active metabolites into the cytoplasm [10].

DM4 binds to tubulin and disrupts microtubule dynamics, leading to cell cycle arrest in the G2/M phase and subsequent apoptotic cell death. The high potency of DM4 enables substantial antitumor activity even with limited intracellular delivery.

In addition, MIRV exhibits a bystander effect, whereby membrane-permeable DM4 metabolites diffuse into neighboring tumor cells, including those with low or heterogeneous FRα expression [27]. This effect may enhance antitumor activity in tumors with intratumoral heterogeneity [27, 28].

## Preclinical rationale underpinning subsequent clinical development

### Preclinical proof-of-concept

Preclinical studies have confirmed that FRα is highly expressed in EOC and can be effectively exploited for targeted drug delivery. MIRV selectively binds to FRα-positive tumor cells and induces cytotoxicity through intracellular delivery of the DM4 payload, demonstrating potent, expression-dependent antitumor activity in preclinical models [10].

MIRV mediates cytotoxicity through microtubule disruption, a mechanism distinct from DNA-damaging agents such as platinum compounds, supporting its activity in platinum-resistant tumors [29]. Consistent with preservation of FRα expression in platinum-resistant disease, MIRV demonstrated efficacy in platinum-resistant models, including patient-derived xenografts, with tumor regression observed in multiple studies [10, 27].

As discussed in Sect. “Cellular uptake, intracellular processing, and cytotoxic effects”, membrane-permeable DM4 metabolites generated from MIRV may induce bystander cytotoxicity in neighboring tumor cells with low FRα expression, potentially enhancing antitumor activity in heterogeneous tumors [10, 27].

Preclinical studies have further demonstrated potential synergy between MIRV and cytotoxic agents, including platinum compounds and anthracyclines [27]. Anti-angiogenic agents such as bevacizumab may also enhance MIRV efficacy by improving drug delivery through vascular normalization [10, 25, 27]. These findings provided a rationale for MIRV-based combination strategies.

### Implications for clinical development

The preclinical data highlight key principles underlying MIRV development, including the importance of FRα expression as a predictive biomarker, its activity in platinum-resistant disease, and its compatibility with combination strategies [10, 27, 30]. Collectively, these findings provided a foundation for subsequent clinical trials and guided MIRV development in ovarian cancer.

## Clinical development of mirvetuximab soravtansine

Major clinical trials leading to MIRV development and regulatory approval are summarized in Table 1. The clinical development of MIRV has highlighted the importance of biomarker-driven therapies for ovarian cancer. Its trajectory, from early-phase studies through an initially negative phase III trial to subsequent success with refined biomarker selection, underscores the critical role of accurate patient selection and diagnostic optimization [11–15].

**Table 1:** Major clinical trials of Mirvetuximab Soravtansine in patients with platinum-resistant ovarian cancer

Clinical Trial ID	NCT01609556	NCT02631876	NCT04296890	NCT04209855
Study name		FORWARD I	SORAYA	MIRASOL
Special notes	• First in human trial • Established recommended dose (6 mg/kg)	A negative trial that highlighted the importance of biomarker selection	Basis for FDA accelerated approval	Confirmatory phase III trial
Phase	Phase I	Phase III	Phase II	Phase III
Patients	Recurrent (n = 46)	Recurrent, FRα-positive (MIRV n = 243; chemotherapy n = 123)	Recurrent, FRα-high (n = 106)	Recurrent, FRα-high (MIRV n = 227; chemotherapy n = 226)
≥ 3 previous lines of therapy	> 50%	34%	51%	46%
Previous PARPi exposure	NR	17.7%	48%	55%
Previous bevacizumab exposure	NR	48.8%	100%	61%
Treatment	MIRV	1. MIRV 2. IC chemotherapy	MIRV	1. MIRV 2. IC chemotherapy
ORR	26.1%	22% vs 12% (NS)	32.4%	42.3% vs 15.9% (*p* < 0.0001)
Complete response	1%	NR	4.8%	5% vs 0%
mDOR	19.1 weeks	NR	6.9 M	NR
mPFS	4.8 M	4.1 M vs 4.4 M [HR 0.98 (0.73–1.31), *p* = 0.897]	5.5 M	5.6 M vs 4.0 M [HR 0.65 (0.52–0.81), *p* < 0.0001]
mOS	NR	16.4 M vs 14.0 M [HR 0.815; *p* = 0.248]	13.8 M	16.5 M vs 12.8 M [HR 0.67 (0.50–0.89), *p* = 0.0046]

FRα, folate receptor alpha; mDOR, median duration of response; NR, not reported; ORR, objective response rate; OS, overall survival; PARPi, poly (ADP-ribose) polymerase inhibitors; M, months; PFS, progression-free survival; OS, overall survival; IC, investigator’s choice; NS, not significant; HR, hazard ratio; FDA, Food and Drug Administration

### Early-phase clinical trials (phase I)

Early-phase studies established the safety, dosing, and preliminary efficacy of MIRV in heavily pretreated, FRα-positive ovarian cancer (NCT01609556). MIRV administered at 6 mg/kg (adjusted ideal body weight) every three weeks demonstrated a manageable toxicity profile, including predominantly gastrointestinal AEs, fatigue, and blurred vision [11].

Encouraging antitumor activity was observed, with an objective response rate (ORR) of approximately 26% overall and up to 39% in less heavily pretreated patients [11], supporting further phase III development.

### The FORWARD I trial: lessons learned

The phase III FORWARD I trial compared MIRV (n = 243) with investigator’s choice chemotherapy (n = 123) in patients with PROC but did not meet the primary endpoint of PFS in the intention-to-treat population (NCT02631876). Median PFS was 4.1 months with MIRV vs 4.4 months with chemotherapy (HR 0.98; 95% CI 0.73–1.31; *p* = 0.897) [12].

However, exploratory analyses suggested improved outcomes in patients with high FRα expression, including higher response rates and better patient-reported outcomes [12]. The negative results were largely attributed to suboptimal biomarker selection and assay limitations.

These findings underscore the importance of precise patient selection and informed the design of subsequent trials restricted to FRα-high populations.

### Pivotal trials leading to regulatory approval

#### SORAYA trial

The phase II SORAYA trial evaluated MIRV monotherapy in patients with FRα-high PROC who had received one to three prior lines of therapy, including prior bevacizumab exposure (NCT04296890). A total of 106 patients were enrolled.

MIRV demonstrated clinically meaningful activity, with an ORR of 32.4% (95% CI 23.6–42.2) and a median duration of response of 6.9 months (95% CI 5.6–9.7) [13, 14]. These results supported the accelerated regulatory approval of MIRV in biomarker-selected populations.

#### MIRASOL trial

The phase III MIRASOL trial confirmed MIRV efficacy in patients with FRα-high PROC, comparing MIRV (n = 227) with investigator’s choice chemotherapy (n = 226).

MIRV significantly improved PFS (median 5.62 months vs 3.98; HR 0.65; 95% CI 0.52–0.81; *p* < 0.001) and OS (median 16.46 months vs 12.75 months; HR 0.67; 95% CI 0.50–0.89; *p* = 0.005) [14]. Additionally, MIRV demonstrated a higher ORR (42.3% vs 15.9%) and a more favorable safety profile than chemotherapy. These findings established MIRV as an important treatment option for patients with FRα-high PROC.

### Clinical determinants of efficacy

Across clinical trials, MIRV has demonstrated consistent antitumor activity in patients with FRα-high PROC, with ORRs of approximately 30–40% and meaningful improvements in PFS and OS compared with chemotherapy [13–15]. High FRα expression is the most important determinant of response, as consistently shown across studies [13–15]. Efficient receptor-mediated internalization and intracellular processing are also critical for effective payload delivery and cytotoxic activity [10].

Despite these clinically meaningful outcomes, a substantial proportion of patients with FRα-high tumors do not respond to MIRV. Potential explanations include intratumoral heterogeneity of FRα expression, insufficient ADC internalization, impaired intracellular trafficking or lysosomal processing, and intrinsic resistance to the DM4 payload. Tumor microenvironmental factors and prior treatment exposure may also influence treatment sensitivity.

Tumor heterogeneity may influence response; however, the bystander effect of MIRV may partially overcome this limitation by enabling cytotoxic activity in adjacent tumor cells with low or heterogeneous FRα expression [10, 27, 23, 28]. Pharmacokinetic factors may also contribute to differential outcomes, with exposure–response analyses suggesting an association between MIRV exposure and clinical efficacy, although dose optimization based on these parameters has not been established [31]. Collectively, these findings underscore both the importance and the limitations of biomarker-driven patient selection and highlight the need for improved predictive biomarkers and resistance characterization.

## Safety profile and management

### Overall safety profile

Across clinical trials, MIRV has demonstrated favorable tolerability compared with chemotherapy. In the phase III MIRASOL trial, MIRV was associated with fewer grade ≥ 3 AEs (41.7% vs 54.1%) and fewer serious AEs [14]. The safety profile is characterized mainly by gastrointestinal symptoms, fatigue, and peripheral neuropathy, typically grades 1–2 and generally manageable with standard supportive care [32]. Integrated safety analyses involving over 600 patients confirmed that MIRV-related toxicities were predictable and associated with low discontinuation rates [32].

### Ocular toxicity

#### Characteristics and mechanism

Ocular AEs are the most characteristic toxicities associated with MIRV, including blurred vision and keratopathy, and are generally low-grade and reversible [31–34]. Approximately 50% of patients experienced ocular events, most grade ≤ 2, while grade ≥ 3 events are uncommon (~ 5%) [31, 32]. These toxicities are attributed to off-target effects of the DM4 payload on the corneal epithelium rather than FRα-mediated mechanisms, as FRα is not expressed in corneal tissues [31, 32].

#### Management and clinical considerations

Ocular toxicity can be managed using prophylactic and supportive measures, including lubrication and corticosteroid eye drops [31, 34]. Baseline and periodic ophthalmologic assessments are recommended, particularly during early treatment cycles. Dose delays or reductions may be required in symptomatic cases; however, most events resolve with appropriate management. Ongoing studies are evaluating optimized prophylactic strategies for ocular AEs (Table 2), including a phase II trial comparing different eye drop regimens (NCT06365853) [35].

**Table 2:** Ongoing Clinical Trials of Mirvetuximab Soravtansine in patients with ovarian cancer

Trial	Phase	Population	Regimen	Notes
1. Maintenance setting				
NCT05445778 (GLORIOSA)	III	Recurrent platinum-sensitive, FRα-high (≥ 75%)	Platinum-based chemotherapy + bevacizumab → MIRV + bevacizumab vs bevacizumab	Maintenance after platinum + bevacizumab
NCT05456685	II	Recurrent platinum-sensitive, FRα-positive	MIRV + carboplatin → MIRV continuation	Induction + maintenance strategy
NCT05887609	II	Recurrent platinum-sensitive	Platinum-based chemotherapy → MIRV + olaparib	PARP-based maintenance after platinum
2. Frontline/early-line setting				
NCT06890338	II	Newly diagnosed, FRα-positive	MIRV + carboplatin	Early-line integration
3. Recurrent disease (efficacy studies)				
NCT05041257 (PICCOLO)	II	Recurrent platinum-sensitive, FRα-high	MIRV monotherapy	Single-agent efficacy study
4. Safety/optimization				
NCT06365853	II	Recurrent (platinum-sensitive or resistant), FRα-high	MIRV monotherapy	Ocular toxicity mitigation study
5. Combination/feasibility setting				
NCT07059845 (FLORENZA)	I/II	Recurrent ovarian cancer (FRα-unselected)	MIRV + carboplatin/bevacizumab	Combination feasibility study

#### Pneumonitis/interstitial lung disease

Although less common than ocular toxicity, MIRV-associated pneumonitis/interstitial lung disease (ILD) has been reported in clinical studies [14, 32]. Most events were low grade; however, rare severe cases have been observed [14, 32]. Severe, life-threatening, or fatal ILD, including pneumonitis, can occur with MIRV; therefore, careful monitoring for respiratory symptoms and radiographic abnormalities is warranted during MIRV treatment [36]. Early recognition, treatment interruption, corticosteroid administration, dose modification, or permanent discontinuation according to severity are important for appropriate management [36].

### Quality of life

MIRV has demonstrated preservation of patient-reported quality of life compared with chemotherapy without meaningful deterioration [13–15, 37].

In the phase III MIRASOL trial, the primary patient-reported endpoint—improvement in abdominal and gastrointestinal symptoms—was not significantly different between MIRV and chemotherapy (21.0% vs 15.3%; *p* = 0.26), although sensitivity analyses suggested greater improvement with MIRV (33% vs 22%; *p* = 0.045) [14, 37]. Exploratory analyses of the EORTC QLQ-C30 showed better global health status, physical and role functioning, and reduced fatigue with MIRV compared with chemotherapy [14, 37], with longitudinal analyses indicating sustained symptom control.

## Expanding indications

MIRV is established as a treatment option for patients with FRα-high PROC. Nonetheless, its clinical application is being explored in earlier disease settings and combination strategies. Ongoing clinical trials evaluating MIRV in various treatment settings are summarized in Table 2.

### Combination with anti-angiogenic therapy

Preclinical studies suggest that combining MIRV with anti-angiogenic agents may enhance antitumor activity through complementary mechanisms, including improved drug delivery via vascular normalization [27]. This rationale was evaluated in the multi-cohort phase Ib/II FORWARD II study (NCT02606305), in which MIRV was combined with agents such as bevacizumab, pembrolizumab, and standard chemotherapy [30, 38]. In the bevacizumab cohort, MIRV demonstrated an ORR of approximately 39% and median PFS of 6.9 months in patients with FR-positive PROC [38]. The phase II expansion cohort reported an ORR of approximately 44% and median PFS of 8.2 months, with a manageable safety profile [30].

Ongoing studies are evaluating MIRV-based combinations, including MIRV with carboplatin and bevacizumab in the phase I/II FLORENZA study (NCT07059845) and maintenance therapy with MIRV plus bevacizumab in the phase III GLORIOSA trial (NCT05445778) [39].

### Platinum-sensitive recurrent ovarian cancer

MIRV is also being investigated for platinum-sensitive recurrent ovarian cancer, where its favorable safety profile supports integration into platinum-based regimens and maintenance strategies. In a phase Ib study combining MIRV with carboplatin and bevacizumab, a high ORR of 83% was observed, with increased hematological toxicity, including thrombocytopenia and neutropenia [40]. These findings highlight both potential efficacy and the need for careful toxicity management in combination regimens.

Ongoing trials, including a phase II study of MIRV combined with carboplatin followed by MIRV continuation (NCT05456685) and the PICCOLO trial (NCT05041257) evaluating MIRV monotherapy in platinum-sensitive recurrent disease [39], will further define its role in this setting.

### Maintenance strategies

MIRV is being explored in maintenance strategies aimed at prolonging disease control following response to platinum-based therapy.

The ongoing phase III GLORIOSA trial (NCT05445778) is evaluating MIRV plus bevacizumab vs bevacizumab alone after platinum-based chemotherapy. Additional studies include a phase II trial incorporating MIRV during induction with carboplatin, followed by MIRV maintenance (NCT05456685), and a phase II trial evaluating MIRV with olaparib as a PARP-based maintenance approach (NCT05887609) [39, 41]. Together, these studies reflect multiple strategies for integrating MIRV into maintenance paradigms.

### Frontline and earlier lines of therapy

There is a strong rationale for introducing MIRV in earlier lines of therapy, where tumor burden is lower and treatment tolerance may improve. Early-phase data suggest higher response rates in less heavily pretreated patients [11].

MIRV is being evaluated in frontline combination regimens, including studies combining MIRV with chemotherapy and targeted agents. Ongoing trials include a phase II study of MIRV plus carboplatin in newly diagnosed ovarian cancer (NCT06890338) [39]. These efforts aim to define the role of MIRV across the treatment continuum of ovarian cancer.

### Emerging FRα-targeted therapies and future landscape

In parallel with MIRV development, several next-generation FRα-targeted ADCs and alternative FRα-directed therapeutic approaches are under investigation. These include novel ADCs incorporating different payloads, linker technologies, and antibody designs aimed at improving efficacy, overcoming resistance, and reducing toxicity [5]. Therefore, the future clinical positioning of MIRV may be influenced not only by its own expanding indications but also by evolving competition from newer FRα-targeted agents with distinct pharmacological and safety profiles. Further advances in biomarker strategies, ADC engineering, and combination approaches are expected to shape the future therapeutic landscape of FRα-targeted therapy in ovarian cancer [5, 42].

## Biomarkers and patient selection

### FRα expression as a predictive biomarker

FRα expression assessed by immunohistochemistry is the primary predictive biomarker for response to MIRV, with higher expression consistently associated with higher response rates and longer PFS in clinical studies [11, 13–15].

The importance of biomarker-driven patient selection was underscored in the phase III FORWARD I trial, in which inclusion of patients with low to moderate FRα expression contributed to failure to meet the primary endpoint [12]. In contrast, subsequent trials, including SORAYA and MIRASOL, restricted enrollment to patients with FRα-high tumors (defined as ≥ 75% of tumor cells with ≥ 2 + staining intensity), resulting in improved efficacy outcomes [13–15].

Standardized companion diagnostic assays, such as the VENTANA FOLR1 assay, have improved consistency of FRα assessment; however, variability in assay performance and interpretation remains a limitation [42]. In addition, spatial and temporal heterogeneity of FRα expression represents an important clinical challenge. Although several studies suggest relative preservation of FRα expression after chemotherapy and at recurrence [8, 21, 22], clinically meaningful heterogeneity between primary and metastatic lesions, as well as lesion-to-lesion variability, may still occur and potentially influence treatment response [30].

Intratumoral heterogeneity may contribute to incomplete responses or resistance despite FRα-high classification. The bystander effect associated with MIRV may partially mitigate this limitation by enabling cytotoxic activity in adjacent tumor cells with lower FRα expression, although the extent to which this compensates for heterogeneous target expression in clinical settings remains uncertain [27, 28].

Collectively, these findings highlight both the clinical importance and the limitations of FRα as a predictive biomarker and support continued efforts to improve biomarker assessment strategies for MIRV-based therapy.

### Other predictive factors and future biomarker development

In addition to FRα expression, several factors may influence response to MIRV. Clinical data suggest that patients with fewer prior lines of therapy may derive greater benefit, potentially reflecting differences in tumor biology or treatment sensitivity [11]. Pharmacokinetic analyses indicate an exposure–response relationship between MIRV levels and clinical outcomes, although these findings have not led to dose optimization strategies [31].

Despite this observation, predictive markers beyond FRα expression remain limited. Tumor heterogeneity and variability in treatment responses highlight the need for refined biomarker strategies.

Future efforts should focus on improving biomarker approaches to identify patients most likely to benefit from MIRV. These include quantitative and spatial assessment of FRα expression, identification of additional predictive markers of response or resistance, and development of non-invasive approaches such as circulating biomarkers and imaging-based assays to enable dynamic monitoring of treatment response and disease heterogeneity [5, 24, 42]. These advances will be critical for optimizing patient selection and maximizing the clinical utility of FRα-targeted therapies.

## Limitations and challenges

Despite the clinical success of MIRV, challenges remain; addressing these issues is essential to further improve outcomes and expand its use.

### Mechanisms of resistance

Primary and acquired resistance to MIRV may arise through multiple mechanisms, including reduced or heterogeneous FRα expression, impaired receptor-mediated internalization, and defective intracellular trafficking or lysosomal processing. In addition, potential resistance mechanisms related to the DM4 payload may include drug efflux and altered microtubule dynamics, based on broader resistance models reported for ADCs and microtubule inhibitor–based therapies [5]. A deeper understanding of these resistance pathways is essential for developing next-generation ADCs and rational combination strategies.

### Treatment sequencing

Although MIRV is established as a treatment option for patients with FRα-high PROC, its optimal positioning within the treatment algorithm remains to be defined. Key questions include whether MIRV should be introduced earlier in the disease course, incorporated into platinum-sensitive settings, or reserved for later lines after multiple prior therapies. The influence of prior treatments, including PARP inhibitors and antiangiogenic agents, on MIRV efficacy and patient selection also remains unclear.

Notably, the proportion of patients previously exposed to PARP inhibitors increased substantially across recent MIRV trials (Table 1), reflecting evolving treatment paradigms and clinical practice in ovarian cancer. Increasing use of PARP inhibitors may alter tumor biology and contribute to selection of biologically aggressive or treatment-refractory tumor clones, potentially influencing subsequent response to MIRV. However, the extent to which prior PARP inhibitor exposure directly affects MIRV sensitivity remains unclear and warrants further investigation.

Ongoing clinical studies of MIRV in earlier-line and combination settings are expected to help define optimal sequencing strategies and clarify the role of MIRV within the evolving therapeutic landscape of ovarian cancer.

### Cost-effectiveness

Model-based analyses suggest that although MIRV provides clinically meaningful gains in survival and quality-adjusted life-years (QALYs), it is unlikely to be cost-effective at the current pricing. In a U.S.-based Markov model, MIRV yielded an incremental gain of approximately 0.90 QALYs at a substantially increased cost, resulting in incremental cost-effectiveness ratios that exceed commonly accepted willingness-to-pay thresholds [43]. These findings indicate that the economic value of MIRV is highly sensitive to drug pricing and model assumptions. Further real-world and region-specific evaluations are necessary to better define its cost-effectiveness and inform reimbursement decisions [13–15].

## Conclusion

MIRV has established a new paradigm of biomarker-driven therapy in PROC, but key challenges remain, including resistance, biomarker limitations, and optimal treatment sequencing. Addressing these will be essential to improve the efficacy and durability of MIRV-based therapies. Future research should refine patient selection beyond FRα expression and identify additional predictive biomarkers, supported by rational combination strategies, advances in ADC technology, and real-world evidence.

These efforts are expected to further integrate MIRV into the treatment continuum of ovarian cancer and advance more personalized therapeutic strategies.

## Abbreviations

ADC: Antibody–drug conjugate
ADCC: Antibody-dependent cellular cytotoxicity
ADCP: Antibody-dependent cellular phagocytosis
AEs: Adverse events
CDC: Complement-dependent cytotoxicity
CI: Confidence interval
DM4: N2′-deacetyl-N2′-(4-mercapto-4-methyl-1-oxopentyl)-maytansine
DNA: Deoxyribonucleic acid
EOC: Epithelial ovarian cancer
FDCs: Folate–drug conjugates
FR: Folate receptor
FRα: Folate receptor alpha
GHS: Global health status
GPI: Glycosylphosphatidylinositol
HR: Hazard ratio
MIRV: Mirvetuximab soravtansine
ORR: Objective response rate
OS: Overall survival
PARP: Poly (ADP-ribose) polymerase
PFS: Progression-free survival
PROC: Platinum-resistant ovarian cancer
QALY: Quality-adjusted life-year
QLQ-C30: European Organization for Research and Treatment of Cancer Quality of Life Questionnaire Core 30
